# Salivary gland microbiome of *Anopheles gambiae*: a mini-review of acquisition, composition, and functional significance

**DOI:** 10.3389/finsc.2026.1911294

**Published:** 2026-08-17

**Authors:** Celestina O. Olafusi, Israel S. Afolabi, Olubanke O. Ogunlana

**Affiliations:** 1 Department of Biochemistry, Covenant University, Ota, Ogun, Nigeria; 2 Covenant Applied Informatics and Communication Africa Centre of Excellence, Covenant University, Ota, Ogun, Nigeria; 3 Department of Biochemistry, University of Medical Sciences, Laje, Ondo, Nigeria

**Keywords:** *Anopheles gambiae*, microbiome, midgut, paratransgenesis, *Plasmodium* infection, salivary gland

## Abstract

The salivary gland (SG) is the final barrier for *Plasmodium* transmission to humans but remains comparatively understudied relative to the midgut microbiome. This review synthesizes current knowledge on SG microbiome acquisition routes, composition, and functional significance. Acquisition may occur via larval filter feeding, vertical (egg smearing), transstadial, or horizontal transmission during blood feeding, though their relative contributions are unknown. Compositional studies show Gram-negative genera *Serratia*, *Elizabethkingia*, *Acinetobacter*, *Pseudomonas*, and *Asaia* predominate; *Plasmodium* infection correlates with increased *Serratia* and decreased *Elizabethkingia* abundance. While immune-related genes (e.g., cecropins, defensin, GNBP, SRPN6) expressed in the SG may be modulated by resident bacteria, direct evidence of their effect on sporozoite invasion remains lacking. Gram-negative bacteria trigger Toll, Imd, and JAK-STAT pathways, but emerging evidence suggests the SG may mount a distinct, locally independent immune response compared to the systemic pathway. Paratransgenesis using *Asaia* shows promise, yet SG-targeted effector delivery remains untested. Ecological pressures common in West Africa, including agricultural pesticides, insecticide resistance, and larval water contamination, may influence mosquito-associated bacteria, but no studies explicitly link these to the SG microbiome. Significant knowledge gaps persist, notably the absence of field studies in high-burden regions like Nigeria and the lack of experimental manipulation to establish causality. Addressing these priorities is critical to determine whether the SG microbiome can be exploited as a transmission-blocking target.

## 1 Introduction

In 2024, an estimated 282 million cases of malaria and 610, 000 deaths were reported, establishing that malaria remains a serious global health challenge, with eleven countries from the WHO region bearing the greatest burden and accounting for approximately two-thirds of global cases and deaths (1). In Africa, Nigeria carries the highest burden of this disease, accounting for approximately 24.3% of the global malaria burden and 31% of malaria deaths (2), with *Anopheles gambiae* being the primary vector driving the ongoing transmission of *Plasmodium falciparum* in the Nigerian population (3). Indoor residual spraying (IRS) and insecticide-treated bed nets (ITNs) are some of the vector control strategies that have historically proven effective in reducing transmission (4, 5); however, resistance to the active ingredients of these interventions has spread among vector populations and has beset these interventions with difficulties (6). Resistance to the most widely used therapies, including artemisinin-based combination therapies, in *Plasmodium* parasites has also been reported (7), underscoring the need to design novel strategies to diminish the parasite-transmitting capacity of the mosquito (8). Using the bacteria that inhabit the mosquito to erect physiological or immunological barriers to infection, whether through physical interference, immune priming, or resource competition, such that the mosquito becomes refractory to *Plasmodium* infection, is a powerful approach (9, 10).

The first site of *Plasmodium* infection is the midgut of the mosquito vector; however, the salivary gland represents the final barrier before transmission to humans (11, 12), as the sporozoites must successfully invade the salivary glands (13). A complex community of microorganisms called the microbiome lives inside the mosquito, and it has been shown to influence the vector’s competence (14, 15). Studies have reported that the salivary gland harbors a distinct bacterial community (11, 16–19), and that these bacteria may influence sporozoite invasion. A study reported *Pseudomonas* and *Acinetobacter* as the predominant species among other bacterial communities that were profiled in the salivary glands (16). Critically, even if a mosquito has a heavy midgut infection, transmission will not occur if the salivary gland microbiome or immune environment blocks sporozoite entry or survival. Existing knowledge on the *An. gambiae* salivary gland microbiome will be synthesized in this review, with a focus on (i) the routes through which these microbial communities are acquired; (ii) the composition of the microbial communities inhabiting the salivary gland, and (iii) the functional significance of the salivary gland microbiome, its interaction with *Plasmodium* development, mosquito immunity, and potential for paratransgenesis.

## 2 Literature search strategy and review methodology

A literature search was carried out to determine the scope of the problem. For this review, completed literature was searched using Scopus, Web of Science, PubMed and Google Scholar with the search terms: (“*Anopheles gambiae*” OR “*Anopheles*”) AND (“salivary gland”) AND (“microbiome” OR “microbiota” OR “bacteria”) AND (“vector competence” OR “immune” OR “*Plasmodium*”) using a lower cut-off year of 1980 and an upper cut-off year of 2026, including the titles, abstracts, and keywords.

Inclusion criteria: (i) peer-reviewed, English-language studies on the salivary gland microbiome of *Anopheles gambiae*; (ii) studies on other *Anopheles* species or mosquito taxa reporting salivary gland microbiome composition, acquisition, or immune-pathway mechanisms directly relevant to *An. gambiae* biology, where *An. gambiae*-specific data were unavailable; (iii) midgut/whole-body microbiome studies in *Anopheles* species with direct relevance to salivary gland colonization or vector competence.

Exclusion criteria: (i) studies not focused on the salivary gland biology of *Anopheles*/mosquito species; (ii) non-English publications; (iii) conference abstracts, theses, or preprints without full text; (iv) duplicate records.

**Figure 1:**
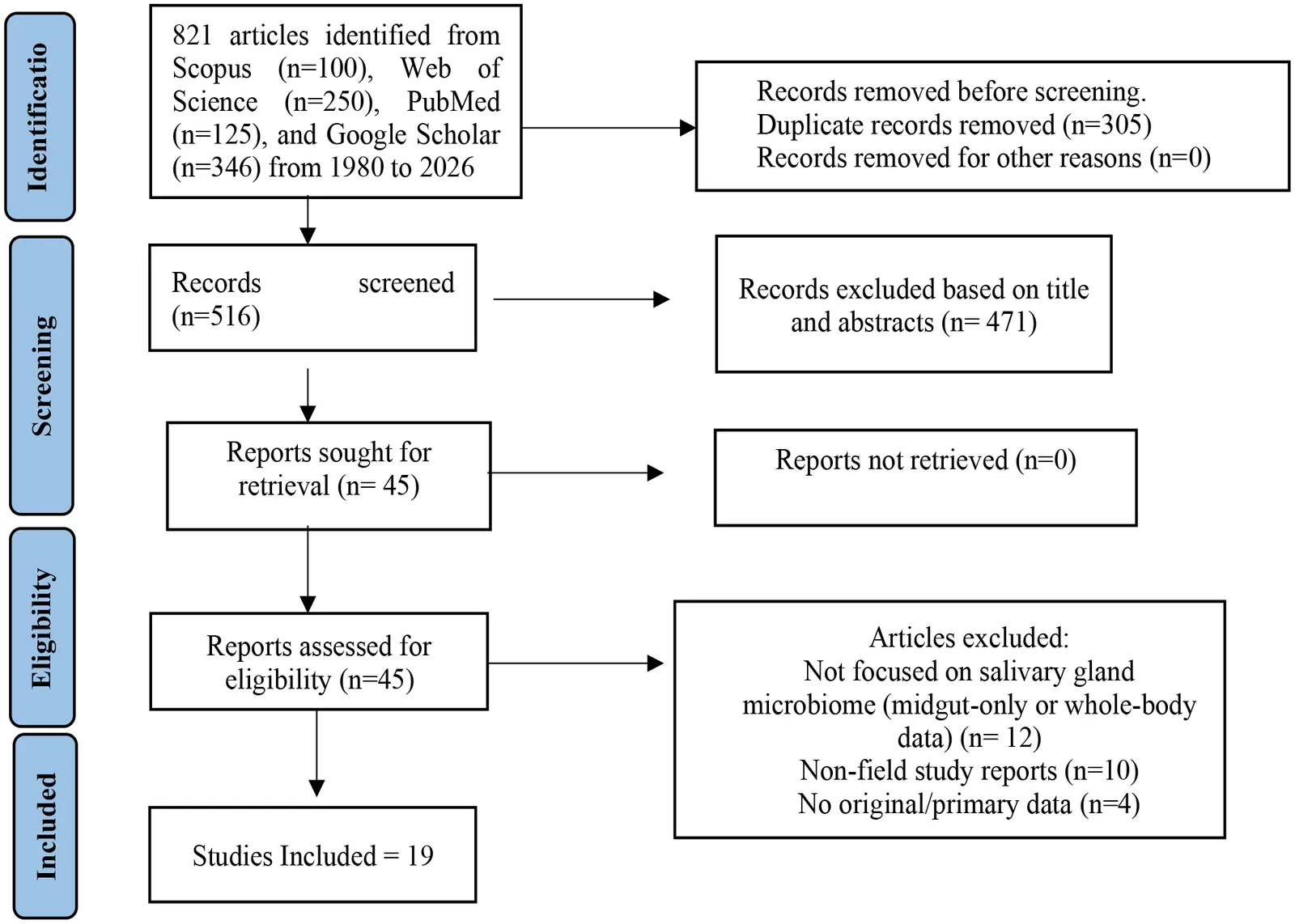
PRISMA 2020 flow diagram of the study selection process.

The search identified 821 articles (Scopus, n = 100; Web of Science, n = 250; PubMed, n = 125; Google Scholar, n = 346). 305 articles were excluded as duplicates, leaving 516 articles for title and abstract screening, of which 471 were excluded for not meeting the inclusion criteria. The remaining 45 articles were evaluated in full-text screening, 19 of which were included in qualitative synthesis after excluding 26 articles that were of background-only relevance, did not provide original data, duplicated an already included dataset, and were not focused on the salivary gland microbiome (**Figure 1**). A priority was given to studies on the salivary gland microbiome that are experimental, mechanistic, field or clinical, and provide insight into the composition, acquisition routes, functional significance, and transmission-blocking potential of this microbiome. The findings were narratively summarized and arranged thematically by acquisition route, microbial composition, and functional significance, which is typical for a mini-review, not a quantitative meta-analysis.

## 3 The salivary gland structure and function

The salivary gland of *An. gambiae* consists of one medial and two lateral lobes, the latter of which is divided into proximal and distal regions (20). However, in the female mosquitoes’ salivary glands, the genes involved in blood-feeding are expressed primarily in the distal-lateral lobes (the same region preferentially invaded by sporozoites) (21). A wide array of secreted compounds delivered in saliva are produced by the salivary gland, and they aid mosquito blood feeding by affecting the host’s hemostatic response. The better-studied components in the saliva of hematophagous arthropods are the anti-hemostatic activities, such as platelet inhibitors, vasodilators, and anticoagulants (22, 23). *Plasmodium* parasites’ invasion of the salivary glands is thought to be mediated via receptor-ligand interactions, with several proteins such as circumsporozoite protein 1 (CSP) and salivary gland surface protein (SGS) being implicated in this process, as seen in **Table 1** (28, 29). Recognition and invasion of the glands is a species-specific process that is essential for transmission and requires interactions between ligand(s) on the parasite surface and receptor(s) (30–32), hence the need to study the salivary gland microbiome based on species.

**Table 1:** Some *An. gambiae* salivary gland genes and their functions.

No.	Gene/protein	Functional category	Biological function	References
1	D7L1/D7L2 (D7 long forms)	Anti-hemostatic	Anti-hemostatic; binds biogenic amines (serotonin, histamine, norepinephrine).	(21, 24, 25)
2	D7 short forms (D7r1–D7r5)	Anti-hemostatic	D7r1 inhibits the plasma contact system.	(21, 24, 25)
3	Anophelin (cE5)	Anticoagulant	α-thrombin inhibitor; prevents blood coagulation.	(21, 25)
4	Peroxidase 5B	Salivary protein	Vasodilator via catechol oxidase activity; peroxidase and cell adhesion; sclerotization.	(21, 24, 25)
5	TCTP (Translationally Controlled Tumor Protein)	Salivary protein	Vasodilator via catechol oxidase activity; peroxidase and cell adhesion; sclerotization.	(21, 24, 25)
6	Peroxinectin	Salivary protein	Vasodilator via catechol oxidase activity; peroxidase and cell adhesion; sclerotization.	(21, 24, 25)
7	Apyrase	Anti-hemostatic	Antiplatelet; decreases upon *Plasmodium* infection, affecting probing time.	(21, 24, 25)
8	30 kDa protein (AAPP)	Anti-hemostatic	Antiplatelet; decreases upon *Plasmodium* infection, affecting probing time.	(21, 24, 25)
9	5′-Nucleotidase (5p_nuc)	Anti-hemostatic	Antiplatelet; decreases upon *Plasmodium* infection, affecting probing time.	(21, 24, 25)
10	Saglin	Parasite interaction	Receptor for *Plasmodium* sporozoite invasion of salivary glands.	(25)
11	Mucins (SG3, gSG10, mucin-like)	Structural	Lubricant for mouthparts and salivary ducts.	(21, 25)
12	Cecropin 2	Antimicrobial	Antimicrobial peptide; strongly induced during early salivary gland invasion.	(25, 26)
13	Cecropin 1	Antimicrobial	Antimicrobial peptide; strongly induced during early salivary gland invasion.	(25, 26)
14	Defensin 1	Antimicrobial	Antimicrobial peptide; strongly induced during early salivary gland invasion.	(25, 26)
15	GNBP	Immune	Pattern recognition receptor; activates immune pathways; upregulated upon infection.	(25, 26)
16	SRPN6 (Serpin 6)	Immune	Serine protease inhibitor; antagonist of *Plasmodium* development; upregulated in salivary glands.	(24, 26)
17	Serpin 9	Immune	Serine protease inhibitor; antagonist of *Plasmodium* development; upregulated in salivary glands.	(24, 26)
18	TEP1	Immune	Complement-like opsonin; involved in parasite killing (present in glands).	(26)
19	PRS1	Parasite-associated	DM9 domain protein; induced throughout infection; expressed in distal lateral lobes.	(26)
20	Peritrophin 1	Structural	Chitin-binding protein; structural role in salivary glands; induced by infection.	(26)
21	gVAG (Antigen 5)	Immune	Defense-related; increased 2-fold upon infection; silencing increases *Plasmodium*.	(21, 24)
22	GSG6	Salivary protein	Secreted salivary protein; decreased upon infection.	(24, 27)
23	Lysozyme 1 & 2	Antimicrobial	Bacteriolytic; present in older glands; prevents microbial growth.	(21, 24)
24	C-type lectin	Digestive/Recognition	Hemagglutinating activity/Sugar digestion.	(21)
25	Galectin	Digestive/Recognition	Hemagglutinating activity/Sugar digestion.	(21)
26	Amylase	Digestive	Hemagglutinating activity/Sugar digestion.	(21)
27	Maltase	Digestive	Hemagglutinating activity/Sugar digestion.	(21)
28	Salivary calreticulin	Anti-hemostatic	Possible anti-thrombotic.	(21)
29	TRIO	Structural	Cytoskeletal organization.	(21, 25)
30	Apolipoprotein D precursor	Metabolism	Lipid metabolism; infection-responsive; detected in older glands.	(24)
31	α2-Macroglobulin (ENSANGP00000029324)	Immune	Protease inhibitor; immune-related; detected in older glands.	(24)

## 4 Acquisition of the *An. gambiae* salivary gland microbiome

### 4.1 Larval acquisition

The aquatic environment of the larval diet is a crucial part of the life cycle, and bacteria are ingested via filter-feeding (33, 34). The bacterial community composition in the water surface microlayer is different from the subsurface water community, the surface microlayer being dominated by Betaproteobacteria and Cytophagia, while Bacilli, Alphaproteobacteria and Actinobacteria are more abundant in the water subsurface where larvae live (34). Some of those environmental bacteria survive metamorphosis and can eventually enter the salivary glands in adults.

### 4.2 Vertical transmission

Another route is mother-to-offspring transmission (35), with fluorescent *in situ* hybridization on the reproductive tract of female *An. gambiae*, revealing that *Asaia* bacteria are concentrated at the periphery of eggs, suggesting that egg-smearing mediates vertical transmission of this genus (36).

### 4.3 Transstadial transfer

Microbial taxa can also carry over between developmental stages (37, 38). Although this transstadial transfer is well documented for the midgut, whether it consistently leads to colonization of the adult salivary glands remains incompletely understood.

### 4.4 Horizontal acquisition via blood feeding

In *An. gambiae*, *Rickettsia felis* bacteria acquired from an infected mouse were subsequently detected in the salivary glands and transmitted to naive mice during subsequent blood feeding (11), demonstrating that pathogens and potentially commensal microbes can be acquired horizontally and colonize the salivary gland. Despite these findings, the relative contribution of each route to the final salivary gland microbiome is unknown. Whether nectar or sugar meals introduce bacteria directly into the salivary glands, and whether the salivary glands harbor a stable, self-sustaining community or are continually reseeded from the midgut or the environment, remain open questions.

## 5 Microbial composition of the *An. gambiae* salivary gland

Literature has revealed different microorganisms present in the salivary gland microbiome of *An. gambiae* as shown in **Table 2** below:

**Table 2:** Microorganisms identified in the salivary gland of *An. gambiae* and their characteristics.

Microorganism	Gram reaction	Phylum	Mosquito source	Infection status	Detection method	References
*Asaia* spp. (e.g., *A. krungthepensis*, *A. bogorensis*)	Gram-negative	Proteobacteria	Lab-reared	Not examined	PCR, culture, TEM, FISH	(36)
*Serratia marcescens*	Gram-negative	Proteobacteria	Lab-reared	Uninfected vs *P. berghei* infected	16S rRNA sequencing, MALDI-TOF, culture	(11)
*Elizabethkingia* spp. (e.g., *E. meningoseptica*/*E. miricola*)	Gram-negative	Bacteroidetes	Lab-reared	Uninfected vs *P. berghei* infected	16S rRNA sequencing, MALDI-TOF, culture	(11)
*Acinetobacter* spp.	Gram-negative	Proteobacteria	Lab-reared	Not examined	16S rRNA sequencing, MALDI-TOF	(11)
*Comamonas* spp.	Gram-negative	Proteobacteria	Lab-reared	Not examined	16S rRNA sequencing	(11)
*Pseudomonas* spp.	Gram-negative	Proteobacteria	Lab-reared	Not examined	16S rRNA sequencing, MALDI-TOF	(11)
*Escherichia–Shigella*	Gram-negative	Proteobacteria	Lab-reared	Not examined	16S rRNA sequencing	(11)
*Klebsiella* spp. (e.g., *K. pneumoniae*)	Gram-negative	Proteobacteria	Field-caught	Not examined	Culture, biochemical tests, VITEK 2	(18)
*Staphylococcus* spp. (e.g., *S. epidermidis*, *S. aureus*)	Gram-positive	Firmicutes	Lab-reared	Not examined	Culture, VITEK 2	(18)
*Streptococcus* spp. (e.g., *S. thoraltensis*, *S. mitis/oralis*)	Gram-positive	Firmicutes	Lab-reared	Not examined	Culture, VITEK 2	(18)
*Enterococcus casseliflavus*	Gram-positive	Firmicutes	Lab-reared	Not examined	Culture, VITEK 2	(18)

## 6 Functional significance of the *An. gambiae* salivary gland microbiome

### 6.1 Immune-related gene expression in the salivary gland

The salivary gland expresses a range of immune-related genes that could be modulated by resident bacteria, as shown in **Table 1**; it is not just a passive site for sporozoite accumulation. 37 immune-related genes were identified in the *An. gambiae* salivary gland using Serial Analysis of Gene Expression (SAGE), of which four (GNBP, Serpin6, Defensin1, and Cecropin2) were upregulated during *Plasmodium berghei* invasion (26). Therefore, there are suggestions that the salivary gland can sense and respond to microbial stimuli, including its own bacterial inhabitants, due to the presence of these antimicrobial peptides and pattern-recognition receptors. Mechanistically, diaminopimelic (DAP)-type peptidoglycan on the bacterial cell wall is a main target of the Immune deficiency (Imd) pathway, which recognizes the peptidoglycan recognition protein PGRPLC and activates the Rel/NF-κB transcription factor REL2 to initiate transcription of antimicrobial peptides such as cecropins and defensins (39). In *An. gambiae*, a recent functional genetic analysis between five bacterial species (*E. coli*, *S. aureus*, *B. thuringiensis*, *E. faecalis*, *L. monocytogenes*) showed that Imd/REL2 is the predominant pathway to resist systemic bacterial infection, even Gram-negative bacterial challenge, while the Toll/REL1 pathway is only involved in highly virulent bacterial infections (40). In fact, the activation of the JAK-STAT pathway has been observed following a bacterial challenge: the nuclear translocation of the STAT orthologue, Ag-STAT, in fat body cells (41). Notably, pathway usage is strain-specific: *Serratia* strain Y1 restricts *Plasmodium* via Toll/REL1, whereas another *Serratia* strain (J1) has no such effect (42), demonstrating that individual Gram-negative strains determine which pathway is engaged.

Whether this immune modulation is local to the salivary gland or driven systemically from the gut has been directly tested for Gram-negative bacteria. Following a local challenge (infectious sugar meal) with *E. coli* (a reference laboratory strain representing a Gram-negative model organism), most salivary transcripts increased between 5 and 24 hours post-challenge. In contrast, following a systemic challenge (thoracic injection) with the same bacterium, cecropin was rapidly induced within 5 hours, consistent with a fat-body/hemocyte systemic response, yet none of the salivary-gland-enriched candidate genes showed any increase at either 5 or 24 hours post-injection (43). However, it is important to note that this experimental *E. coli* challenge strain is taxonomically different, but very closely related, to the naturally colonizing *Escherichia-Shigella* found in salivary gland compositional surveys of wild *An. gambiae* (Section 5) and that the functional immune data discussed here are from experimental challenge with a laboratory reference strain and not a field-isolated colonizing bacterium. This dissociation indicates that local Gram-negative challenge triggers an intrinsic salivary gland response independent of the systemic response and possibly locally gated. This local capacity is functionally supported by the salivary peptide hyp6.2, whose transcript is induced by local bacterial challenge and which directly inhibits *Serratia marcescens in vitro* (44). Thus, the salivary gland is immune-competent locally in a Gram-negative-responsive fashion, and this local response is distinct from the canonical systemic Imd/Rel2 response. However, studies have not yet identified which specific pathway (Imd, Toll, or JAK-STAT) is predominantly engaged by either *Serratia* or *Elizabethkingia* in the salivary gland; however, previous studies have identified *Serratia* and *Elizabethkingia* to be particularly predominant in the midgut and reproductive tissues (45).

### 6.2 Direct evidence for bacterial interaction with *Plasmodium* in the salivary gland

The saliva microbiome of *An. gambiae* was characterized using 16S rRNA sequencing and MALDI-TOF (11). Bacteria were demonstrated to be present in mosquito saliva using a transgenic fluorescent strain of *Serratia marcescens*, and these bacteria are transferred to a mammalian host during blood feeding, hence can colonize mammalian tissues. It was shown that *Plasmodium berghei* infection modified the salivary gland microbiota, with the abundance of *Serratia* increasing while that of *Elizabethkingia* decreasing. In addition to this, both *P. berghei* and *Serratia* were co-transferred and colonized mammalian tissues, although the study did not establish whether sporozoite invasion was promoted or inhibited.

### 6.3 Sporozoite invasion as a target for microbial interference

The salivary gland epithelium must be successfully invaded by *Plasmodium* sporozoites for transmission to occur. An immune-inducible serine protease (SRPN6) was reported as a midgut invasion marker but is also specifically induced in salivary glands with the onset of sporozoite invasion (20), which localizes to the basal region of epithelial cells in proximity to invading sporozoites. The number of sporozoites present in the salivary gland was significantly increased upon the knockdown of SRPN6 during the late phase of sporogony, with no effect on oocyst rupture, demonstrating that SRPN6 actively limits sporozoite accumulation.

### 6.4 Paratransgenesis: engineering the salivary gland microbiome

One promising strategy is the genetic modification of symbiotic bacteria to deliver anti-pathogen effectors (paratransgenesis), and one strong candidate for salivary gland-targeted paratransgenesis is *Asaia*. Asaia was isolated from laboratory-reared and wild colonies of *An. gambiae*, detected by PCR in all developmental stages, and in all specimens that were analyzed, it was shown to be localized in the midgut, salivary glands, and reproductive organs. It was reported that *Asaia* had the ability to colonize *An. gambiae* mosquitoes, using recombinant strains of *Asaia* expressing fluorescent proteins (36), suggesting that *Asaia* has potential for use in the paratransgenic control of malaria transmitted by *An. gambiae*.

### 6.5 Ecological and environmental drivers of salivary gland microbiome diversity in West Africa

Agricultural pesticide use in West Africa may exert selective pressures on mosquito populations with downstream effects on the salivary gland microbiome. Insecticide resistance, widespread in West African *An. gambiae*, imposes physiological trade-offs that can compromise immune function and alter bacterial regulation in mosquito tissues, including the salivary glands (46, 47). Contamination of larval breeding water with organic pollutants, common in peri-urban and rural settings of West Africa, shapes the bacterial communities available for larval acquisition (38). In southern Ghana, Anopheles-positive breeding habitats harbored distinct bacterial profiles dominated by *Gammaproteobacteria* and *Betaproteobacteria*, and larvae acquired these same taxa from their breeding water (48). This means that environmental water quality directly influences the microbial pool that can be passed through metamorphosis to adult stages. A field study in West Africa revealed that the collection site was the key factor contributing to the bacterial diversity and accounted for about 20% of the variation, whereas species of mosquitoes and blood meal, and insecticide resistance genotype did not significantly explain the variation in bacterial composition (49). Therefore, local factors including exposure to pesticides, breeding water quality, and other environmental factors are probably the most important factors shaping mosquito-associated bacterial communities and potentially the salivary gland microbiome. Although these likely associations have been made, none of these environmental factors have been directly linked to the microbiome of the salivary glands of wild *An. gambiae* from West Africa. This is a critical knowledge gap that is especially relevant in Nigeria, where there could be specific ecological conditions that result in different compositions of the microbiome in the salivary glands. There is an urgent need for future environmental metadata-focused field studies to integrate salivary gland profiling.

## 7 Gaps

In contrast to the midgut microbiome, there has been much less work done to study the salivary gland microbiome of *An. gambiae*; there are also some reports that the recognition and invasion of the salivary glands is a species-specific process that is essential for transmission and requires interactions between ligand(s) on the parasite surface with receptor(s) (30–32). Several points of interest emerge from this review and have implications for the development of transmission-blocking strategies. First, acquisition routes (larval, vertical, transstadial and horizontal) are each separately documented, but their relative contribution to the adult salivary gland community is unknown, and no study has looked at a bacterial strain traced from larval ingestion to salivary gland colonization. Second, the few available studies show that *Serratia*, *Elizabethkingia*, *Acinetobacter*, *Pseudomonas* and *Asaia* are associated with the salivary gland, with *Plasmodium* infection associated with an increase in *Serratia* abundance and a *decrease* in Elizabethkingia (11, 36), but whether this change is due to infection or a result of it is not known. Third, while resident bacteria, as well as expressing immune genes that could be modulated, have been found in the salivary gland, there is a lack of direct evidence that they inhibit or promote sporozoite invasion by *An. gambiae*. Fourth, paratransgenesis with *Asaia* is promising (36); however, there is no engineered strain that has been tested for anti-sporozoite effector delivery in the salivary gland of *An. gambiae* mosquitoes. Last, it is not yet known whether PGRPLC-induced immune modulation in the SG is a local or systemic response, which can be differentiated by tissue-specific silencing of PGRPLC or REL2 or JAK-STAT pathway components in gnotobiotic *An. gambiae* colonized with specific (defined) strains of *Serrati*a or *Elizabethkingia*; this would be resolved by such experiments. One major gap in the current literature is that field studies of the salivary gland microbiome of wild populations of *An. gambiae* in Nigeria are almost non-existent, where insecticide resistance and transmission intensity could affect the microbiome in ways that are not seen in laboratory colonies.

## 8 Conclusion

A distinct bacterial community is present in the salivary gland of *Anopheles gambiae*, which is acquired via several routes and is dominated by Gram-negative genera such as *Serratia*, *Elizabethkingia*, *Asaia* and *Pseudomonas*, the composition of which is significantly affected by *Plasmodium* infection. The fact that immune genes are found in the salivary gland has led to speculation that resident bacteria might affect local immunity to their host, but there is little direct evidence that they inhibit or promote invasion by sporozoites. The potential of salivary gland-targeted transmission blocking via paratransgenesis with *Asaia* is promising, but yet to be tested. The gaps that need to be filled will ultimately decide whether or not the salivary gland microbiome can be leveraged as a last barrier in malaria control in places such as Nigeria.
